# Late term tolerance in head neck cancer patients irradiated in the IMRT era

**DOI:** 10.1186/1748-717X-8-259

**Published:** 2013-11-05

**Authors:** Gabriela Studer, Claudia Linsenmeier, Oliver Riesterer, Yousef Najafi, Michelle Brown, Bita Yousefi, Marius Bredell, Gerhard Huber, Stephan Schmid, Stephan Studer, Roger Zwahlen, Tamara Rordorf, Christoph Glanzmann

**Affiliations:** 1 Department of Radiation Oncology, University Hospital Zurich, Raemistrasse 100, 8091 Zurich, CH, Switzerland; 2 Clinic for Oral and Maxillofacial, Surgery, University Hospital Zurich, Zurich, Switzerland; 3 Department of Otorhinolaryngology, Head and Neck Surgery, University Hospital Zurich, Zurich, Switzerland; 4 Otorhinolaryngology, Klinik Bethanien, Toblerstrasse 51, 8044 Zurich, Switzerland; 5 Discipline of Oral & Maxillofacial Surgery, Faculty of Dentistry, The University of Hong Kong, Hong Kong, SAR, China; 6 Department of Medical Oncology, University Hospital Zurich, Raemistrasse 100, 8091 Zurich, Switzerland

**Keywords:** Persistent late term effects, Late term tolerance, IMRT and late term effects, Grade 3/4 late effects, IMRT tolerance

## Abstract

**Background:**

The aim was to quantify severe transient and persisting late term effects in our single institution head neck cancer (HNC) cohort treated with curatively intended intensity modulated radiation therapy (IMRT). Hypothesis was if a 2-year follow up (FU) is sufficient to estimate the long term tolerance in HNC irradiated in the IMRT era.

**Methods:**

Between 01/2002-8/2012, 707/1211 (58%) consecutively treated IMRT patients met the inclusion criteria of a FU time >12 months and loco-regional disease control (LRC). 45% presented with loco-regionally advanced disease; 55% were referred for curative definitive IMRT (66 Gy-72 Gy in 30–35 fractions), 45% underwent postoperative IMRT (60-66 Gy in 30–33 fractions). Systemic concomitant therapy was administered in 85%. Highly consistent treatment procedures were performed with respect to contouring processes, dose constraints, radiation schedules, and the use of systemic therapy. Grade 3/4 late term effects were prospectively assessed and analyzed with respect to subgroups at particular risk for specific late effects.

**Results:**

Mean/median FU of the cohort was 41/35 months (15–124). 13% of the patients (92/707) experienced any grade 3/4 late effects (101 events in 92/707 patients), 81% in the first 12 months after radiation. 4% of all developed persisting late grade 3/4 effects (25 events in 25/707 patients).

**Conclusions:**

IMRT led to a high late term tolerance in loco-regionally disease free HNC patients. The onset of any G3/4 effects showed a plateau at 2 years. The question of the cervical vessel tolerance in disease free long time survivors is still open and currently under evaluation at our institution.

## Background

Intensity modulated radiation (IMRT) techniques are expected to improve the therapeutic index for head neck cancer (HNC) by limiting the dose to critical organs and possibly increasing loco-regional tumor control. The benefit of normal tissue sparing achievable with IMRT with resulting lower rates of late term effects in HNC survivors is broadly recognized. Data on successful parotid gland sparing, reduction of radio-osteonecrosis (RON) and dysphagia are published [1-7]. The probability of specific late effects depends on the exposed tissue and on the dose, and sometimes on the chemotherapy.

Aim of this prospectively assessed single center data analysis was to evaluate transient and persisting IMRT late term effects related to subgroups at risk. Hypothesis was if a 2-year follow up (FU) is sufficient to estimate the long term tolerance in HNC patients irradiated in the IMRT era.

## Methods

### Patients

Between 01/2002-8/2012, 1211 HNC patients were consecutively treated with IMRT (or volumetric modulated arc therapy, VMAT) at our department. 707/1211 patients (58%) met the inclusion criteria of a FU time >12 months and loco-regional disease control (LRC), Table 1. 45% presented with advanced disease (T3/4 and/or N2c/N3), 55% were referred for curative definitive IMRT (66 Gy-72 Gy, 30–35 daily fractions), 45% underwent postoperative radiation (60-66 Gy, 30–33 daily fractions). Systemic therapy was administered in 85%. Seven LRC patients were lost after 4.3-10.4 months of FU (median 5.5); 12/707 (2%) LRC patients were lost after 12.2-51.6 months (median 39).

**Table 1:** Demographic and tumor characteristic

**Parameters**	**LRC, >12mo FU**
N patients	707
**Gender** (f: m)	25%:75%
**Mean age** (range)	62 (39-91) years
**Mean/Median FU** (range)	41/35 (15-124) months
**Diagnosis**	
Unknown	19 (3%)
Central oropharynx	96 (14%)
Lateral oropharynx	149 (21%)
Hypopharynx	69 (10%)
Oral cavity	111 (16%)
Nasopharynx	51 (7%)
Larynx	90 (13%)
Sinonasal	40 (5%)
Parotid gland	36 (5%)
Skin	33 (5%)
Nasal	10 (1%)
Others	3 (3%)
**Treatment (dose)**	
Primary IMRT (66-72 Gy, 2.0-2.2 Gy/f)	55% (n=388)
Postoperative IMRT (60-66 Gy, 2.0 Gy/f)	45% (n=319)
**T**	
1	17%
2	34%
3	20%
4	26%
Unknown	3%
**N stage**	
Recurrence	2%
NO	32%
N1-2b	45%
N2c	17%
N3	4%
**Total gross tumor volume (n=388)**	
Mean (range)	62 cc (1-217)
1-15 cc	115
16-70 cc	202
71-130 cc	63
>130 cc	8
**Conc. systemic therapy**	
None	15%
Cisplatin only	62%
Cetuximab only	16%
Cisplatin switched to Cetuximab	7%
**Induction chemotherapy**	3%

In 26 of the 504 excluded patients with locoregional disease (1211–707), G3/4 late effects were assessed (5%), however this information is of limited value as tumor-related symptoms are not always reliably to disdinguish from treatment-related effects, survival time in patients with disease is shorter, FU of patients undergoing palliative systemic therapy often no longer regularly performed by ENT and maxillofacial surgery joint clinics.

Although acute toxicity was not focus of this work, the following characteristics of the study population may be of some interest: early skin tolerance was obviously better than in the pre-IMRT era (no quantitative comparison data) and has been published elsewhere [8]. The percentage of patients needing a PEG insertion to support or replace oral nutrition (46% in the study population) may serve as a surrogate for acceptable dysphagia and mucositis. In addition, no patient had to interrupt his radiation course due to early radiation related side effects.

### Methods

Highly consistent treatment procedures were performed with respect to contouring processes, dose constraints, radiation schedules, use of systemic therapy. Contours and treatment plans were always evaluated by the same two staff radiation oncologists (CG/GS).

Implemented changes as evolved from medical and technical progress over the study time period of 10 years were the following: use of cetuximab in patients with contraindications for cisplatinum (since 04/2006); slight tumor-volume based dose prescription adaptations implemented in 2007 (100% dose coverage of primary GTV in cases with tumor volume >15 cc); clinical implementation of VMAT in 4/2010.

Since 2010, the dose to the brachial plexus has been kept below Dmax 66 Gy, according to the suggestion in most RTOG protocols (http://www.rtog.org/CoreLab/ContouringAtlases/BrachialPlexusContouringAtlas.aspx).

Late normal tissue effects were graded according to the Radiation Therapy Oncology Group (RTOG)/European Organization for Research and Treatment of Cancer (EORTC) radiation morbidity scoring criteria [2].

There are several systems aiming to grade RON. We used the system proposed by Glanzmann and Graetz [9]:

1 Exposed bone without signs of infection for at least 3 months

2 Exposed bone with signs of infection or sequester, but not grades 3–5

3 RON, treated with mandibular resection, with satisfactory result

4 RON with persistent problems despite mandibular resection

5 Death due to RON

The advantage of this classification vs. that of EORTC (LENT/SOMA) or NCI [10] is its connection to therapeutic clinical consequences; nevertheless, grade 2 events mutually correspond in all of the above named classification systems. Most frequent therapy for G2 was limited surgery (partial decortication or debridement, removal of sequesters); these surgical procedures are not defined in our grading system, and were counted as 'G2-3’.

All but the above mentioned 19 lost patients are in regular FU; nearly all (99%) are followed at our HN or maxillofacial surgery joint center clinics at the hospital. Routine tests included, besides the history, physical examination and endoscopy of the pharyngeal–laryngeal region. If these tests showed no evidence of disease, usually no further tests were done but a computed tomography scan (CT) or positron emission tomography (PET)–CT or magnetic resonance imaging at 1 year post-treatment in the majority of patients.

No specific functional swallowing testing was performed to assess subtle (G1-2) dysphagia; reasons to do so were: no baseline examinations available; many patients would have shown mild pathological swallowing findings of no significant impact on the final post treatment function; as we focused on high grade late term effects (G3/4) relevant dysphagia with consecutive swallowing problems (aspiration, aspiration pneumonia, difficulty swallowing solid food, weight loss, need of PEG) is supposed to reliably get communicated by patients themselves and/or diagnosed in the regular FU program in our joint center clinics.

Similarly, no specific tests were performed to assess xerostomia G3/4 but patients’ spontaneous complaints and/or suggestive interview questions in regular FU visits including objective clinical assessment of severe oral mucosal dryness. When we implemented IMRT in the routine, swallowing dysfunction and xerostomia were graded in the first 100 IMRT patients using subjective patient-reported (EORTC head-and-neck 35-item swallowing and aspiration (QLQ-H&N35) quality-of-life (QOL) questionnaire) and objective observer-assessed instruments. As subjective estimations were well fitting with objective assessments in routinely clinical FU interviews [4], we did not continue to use the EORTC subjective estimation questionnaires for assessing clinical FU.

With respect to Lhermittes’ sign, no systematic specific assessments have been performed, which may likely would have revealed more events; however as this symptome is known for transient and spontaneous entire healing without any therapy, this is not of clinical consequence.

No case of brachial plexus neuropathy (BPN) with clinically evident dysfunction (grade >2) was diagnosed in our cohort (patients with post-IMRT neck dissection excluded); mild neuropathy (G1/2) may be missed without specific neurological examination.

### Radiation therapy

In >90% of the cohort the pre-treatment standard imaging modality was PET–CT (mostly with intravenous contrast agent), + tomography (CT or MRI). Planning CT images (2 mm slice thickness) were acquired from the vertex or top of the orbita to the carina with contrast agent infusion in all eligible patients.

Gross tumor volume (GTV) delineation of all patients was based on physical examination and endoscopy as well as on diagnostic preoperative MRI, CT and PET. Contours and treatment plans were always evaluated by the same two staff radiation oncologists (CG/GS), in most cases also by a third staff physician.

Technique: Treatment plans were calculated by the Varian Treatment Planning System (Eclipse® External Beam Planning System, Version 7.3.10 and PRO 8.9, AAA 8.9, Varian Medical Systems).

We used an extended-field SIB-IMRT technique, where the primary tumor is covered in one phase along with the regional lymph nodes by a 6MV dynamic MLC system (Varian Medical Systems, Palo Alto, CA) using a sliding window technique (or using VMAT, since 04/2010). Patients were immobilized from head to shoulders with commercially available thermoplastic masks in the supine position.

Target volumes were delineated as follows: GTV included the gross extent of the primary disease and involved lymph node metastases, taking clinical and radiological findings into account; planning target volume 1 (PTV1) was defined by adding a (5–) 10–20 mm margin to the GTV, dependent on the GTV proximity to critical structures (tight margins mainly if proximity of the GTV to the mandible bone, spinal cord or brachial plexus/CNS, generous margins towards tongue and pharyngeal wall, or in cases with difficult identification of the GTV, or if clinical findings not entirely represented in the available imaging); PTV2 covered areas considered at high risk for potential microscopic disease; PTV3 included the clinically negative cervical lymphatic pathways (elective PTV coverage).

SIB IMRT was performed using the following schedules:

– SIB2.00: Daily dose 2.00 Gy (PTV1)/1.70 Gy (PTV2)/1.54 Gy (PTV3) to a total dose of 70.00 Gy (5 fractions/week).

– SIB2.11: Daily dose 2.11 Gy (PTV1)/1.80 Gy (PTV2)/1.64 Gy (PTV3) to a total dose of 69.60 Gy (5 fractions/week).

– SIB2.2: Daily dose 2.2 Gy (PTV1)/2.0 Gy (PTV2)/1.64 Gy (PTV3) to a total dose of 66.0 Gy (5 fractions/week).

The dose was normalized to the mean dose in PTV1. The prescribed dose encompassed at least 95% of the PTV. In cases with central nervous system involvement, Dmax accepted was 2.00 Gy, to a Dmax of 70.0 Gy total dose (PTV1 = GTV; no margin).

No more than 20% of any PTV received >110% of its prescribed dose, whilst no more than 1% of any PTV received <93% of the prescribed dose. Hundred% of the prescription dose included the primary GTV in patients with a primary tumor volume >15 cc (since ~ 2007).

Our interdisciplinary in-house guidelines recommend an elective neck dissection in patients with initial nodal metastasis >3 cm.

### Chemotherapy

Cisplatin was given in weekly doses of 40 mg/m2 at 1 day a week. Since 04/2006, cetuximab was used in patients with contra-indications for concomitant standard cisplatin chemotherapy (400 mg/m2 loading dose, followed by 250 mg/m2 at 1 day a week).

### Follow up

3–6 weeks after completion of IMRT, all patients were also regularly seen in our joint clinic at the Department of Head and Neck or Maxillofacial Surgery. Institutional standards for patient assessment included physical examination with additional flexible fiber-optic endoscopy approximately every 2 months in the first year of follow-up, every 3 months in the second to third year and every 6 months in the fourth to fifth year.

### Statistics

Kaplan Meier survival curves were performed using the statistics program implemented in StatView® (Version 4.5). P values < 0.05 were considered statistically significant. Unpaired T test and Mann Whitney U test was used to calculate the influence of the tumor volume on late tolerance.

## Results

### Disease control

**Figure 1:**
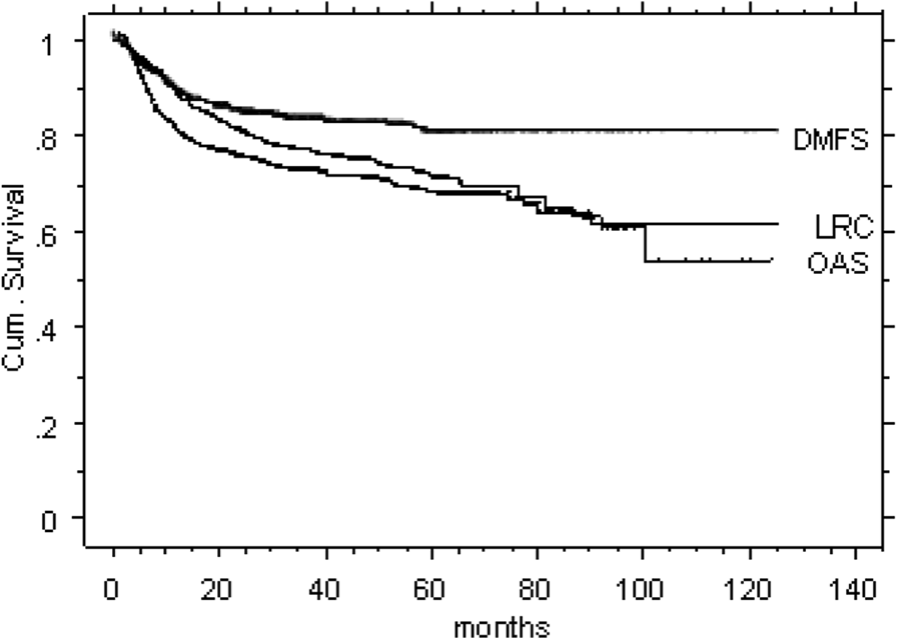
Survival rates of the entire IMRT cohort (OAS: overall survival; LRC: loco-regional control; DMFS: distant metastases free survival).

823 of 1211 (68%) curatively irradiated HNC patients were alive with no evidence of disease when last seen; 14% died from disease, 7% died for other than HNC reasons, 11% were alive with disease. Kaplan Meier survival rates of the 5-year overall survival (OAS, 74%), LRC (68%), and distant metastasis free survival probability (DMFS, 82%) of the entire IMRT cohort are shown in Figure 1.

**Figure 2:**
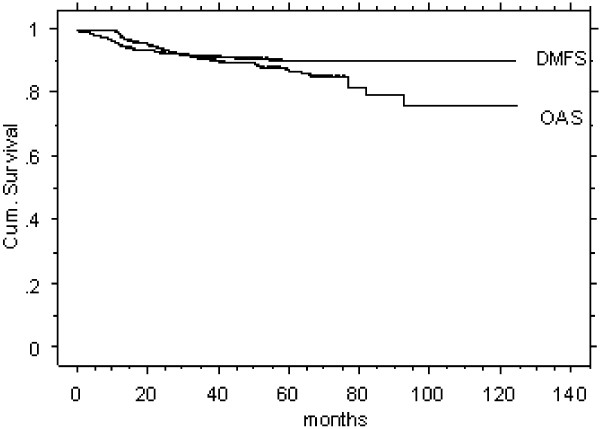
Survival rates of 707 patients assessed for late term tolerance.

707/1211 patients (58%) met the study inclusion criteria of a FU time >12 months and loco-regional disease control (LRC), Table 1. The mean/median FU time of this LRC study cohort was 41/35 months (range 15–124). Five-year DMFS (91%) and OAS (88%) Kaplan Meier curves of these patients are illustrated in Figure 2.

### Late term tolerance

The following late term G3-4 effects were diagnosed (Table 2), Additional file 1: Table S1.

– RON (grade 2–4, [9])

– Mucosal ulcer (other than uncovered bone)

– prolonged (>6 months) need of percutaneous endoscopic gastrostomy (PEG)

– persisting xerostomia/dryness of the pharynx

– need of post-treatment tracheostomy without tumor

– need of post-treatment laryngectomy without tumor

– taste loss >6 months

– Lhermittes’ sign

**Table 2:** Late term effects related to diagnosis

**Parameters**	**RON G2-4**	**Mucosal ulcer**	**PEG**	**Xerostomia**	**Tracheostomy**	**Laryngectomy**	**Loss of taste**	**Others**	**All events/affected patients**
Diagnosis (n), % persistent	lat oro (6/149), 1%	lat oro (9/149), <1%	lat oro (7/149), 0%	lat oro (1/149), 0%	glottic (1/59), 2%	SGL (2/31), 6%	lat oro (1/149), <1%	lat oro (1/149), 0%	
	cent oro (5/96), 0%	centr oro (3/96), 1%	centr oro (8/96), 3%	hypo (1/69), 0%	hypo (1/69), 1%		hypo (1/69), 0%	centr oro (3/96), 0%	
	OCC (10/111), 3%	OCC (8/111), 0%	OCC (4/111), 1%	NPC (3/51), 6%			parotid (2/36), 6%	OCC (1/111), <1%	
		hypo (4/69), 0%	hypo (5/69), 1%					(sino-)nasal (5/50), 5%	
								NPC (1/51), 0%	
All	21/356, 6% G2-4	28/593, 5%	28/606, 5%	5/476, 1%	2/159, 1%	2/159, 1%	4/512, 2%	7/707, 1%	101 events in 92/707 patients, 13%
Persistent G3/4 effects (n)	1% (5)	<1% (2)	<1% (5)	<1% (3)	1% (2)	1% (2)	<1% (3)	<1% (3)	4% (25 events in 25/707 patients)

**Figure 3:**
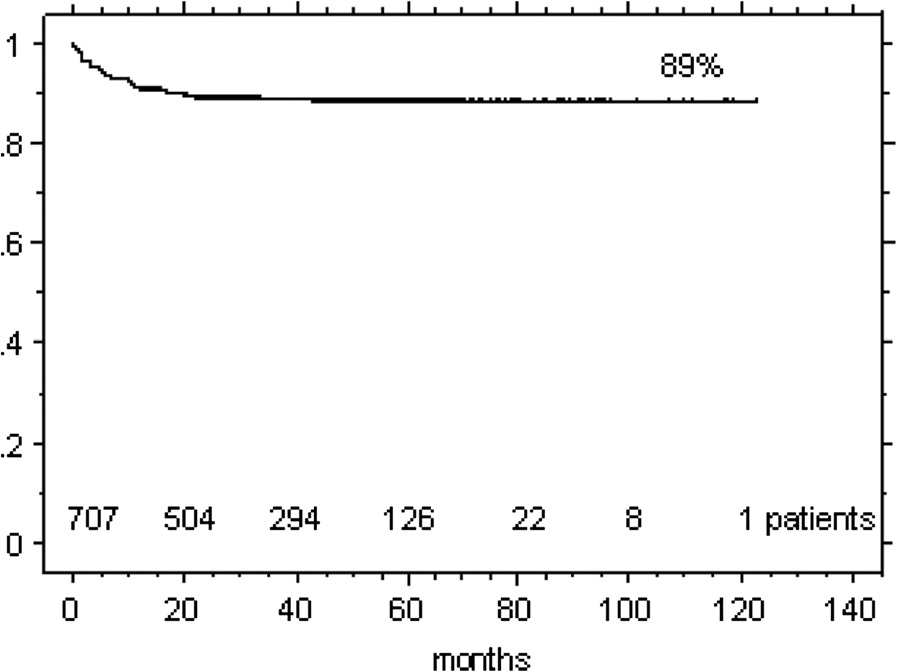
Onset of any G3/4 late effects following IMRT.

The onset of all late term effects (Figure 3) revealed a plateau at 2 years; 81% of all G3/4 events were diagnosed/symptomatic in the first 12 months post radiation. Table 2 (see also Additional file 1: Table S1) shows diagnosed grade 3/4 effects: 92/707 patients (13%) experienced any late term effects, most of them with transient character or complete recovery following specific therapy.

In 4% (25/707), persisting grade 3/4 effects were stated, including:

– two patients with arterial bleeding, one with lethal exit (GTV mean/max 71.8/75.8 Gy, PTV1 mean/max 69.7/76.2 Gy; 2.2 cc/6.5 cc of the GTV/PTV exposed to 74-75 Gy)

– 5 patient with partial mandibulectomy/persisting problems after RON therapy (Table 3); (doses to the RON area of the mandible were <70.0 Gy in 3 patients, 72 Gy point dose in one, 70–72.3 Gy to 1.5 cc in one)

– 5/28 patients with PEG dependence >6 months remained PEG dependent (severe dysphagia +/- aspirations in 4, in one due to insufficient ability to keep his body weight by oral nutrition despite of normal swallowing ability); initially in 46% of all study patients a PEG was inserted to support or replace oral nutrition

– 3 NPC patients with persisting G3 xerostomia (<26 Gy to 62%/36%, 72%/44%, and 48%/52% to right/left parotid glands)

– 2 disease free patients with tracheostomy inserted post IMRT due to edema,

– 1 patient with persisting secretion from a fistula after removal of tracheostoma,

– 2 LRC patients with post IMRT laryngectomy due to necrosis/edema

– 3 patients with insufficient taste recovery,

– one LRC patient with persisting mucosal ulcer after post IMRT biopsy

– one patient with slowly healing oral mucositis (fibrinous mucosal layers >6 months present after 60 Gy postoperative IMRT + concomitant cisplatin)

– 5 nasal/sinonasal tumor patients with persisting repetitive nasal crusts and/or surgical nasal synechiolysis +/- lacrimal duct stenosis stenting

**Table 3:** Grade 1-4 RON in 21/356 patients at risk (oropharynx or oral cavity tumors irradiated with >60Gy to the mandible): 5 with grade 3/4 RON, 3/5 following invasive procedures post-IMRT

**RON, initial G**	**RON therapy (n)**	**Outcome(n): G**	**Complications(n) following RON therapy: final G**	**Onset post IMRT months**	**IMRT sequence**
G1-2	no therapy, spontaneous healing (2)	G0	None: G0	5/21	2 definitive
	debridment + antibiotics (4)*	ad integrum healing (4/4): G0	None: G0	5/6/12	3 postop, 1 definitive
G2-3	partial decortication (11)**	ad integrum healing (10/11)**: G0	pathol. fracture, chronic infection (1/11): G4	3 (0-12)	6 postop, 5 definitive
G3	partial mandibulectomy (4)	ad integrum healing (1): G3	pathol. fracture (1/4) ***: G4	6/6/18/28***	3 postop***, 1 definitive
			osteomyelitis (1/4) ***: G4		
			osteocutaneous fistula (1/4)***: G4		

*: 2/4 with several previous reconstructive soft tissue operations, dental implants with consecutive bone fracture, followed by superficial RON.

**: 2/11 RON following tooth extraction post-IMRT, 1/11 operated patient with pre-IMRT wound revisions/soft tissue debridement, 1/11 with RON following pre-IMRT dental care tooth extraction.

***: 3/4 RON following dental implants and/or other reconstructive surgery post-IMRT.

All but 2/25 individuals with persisting late effects presented with advanced HNC; most with personal history of relevant co-morbidities in addition to nicotine and alcohol abuse.

Tumor volume didn’t show to be a statistically significant predictor for G3/4 late effects in the LRC study group: total GTV (tGTV) in study patients with any G3/4 effects compared with patients with no relevant late effects was mean/median (range) 41/33 (1-132 cc) vs 36/27 (1–217), p = 0.13). The tGTV was expectedly highly significantly larger in non-study patients (with local or nodal failure) as compared with the LRC study cohort: mean/median (range) 55/43 (1-319 cc) vs 39/28 (1-267 cc), p <0.0001.

## Discussion

### Limitations of this study

a) Methodical limitation given by the inclusion criterion of loco-regional disease control: as late term sequelae cannot properly be assessed in patients with persistent or recurrent tumor, locoregional control was a key condition for this evaluation, however, thereby excluding cases with larger tumors and advanced stages, which may be prone to more treatment related late toxicity.

b) The authors are aware of the fact of a still relatively short FU, baring the potential of further upcoming late sequels (e.g. vascular changes)

c) during the study time period of 10 years, several changes due to progresses in medicine and technology have been clinically implemented, like the use of cetuximab 2006, slight own data driven tumor-volume based dose prescription adaptations implemented in 2007, or clinical implementation of VMAT in 2010. These innovations may have influenced treatment tolerance. In addition, since 2010, the dose to the brachial plexus has been kept below Dmax 66 Gy according to the suggestion in most RTOG protocols. Of potential benefit with respect to late tolerance may be our risk adapted pre-IMRT dental care (DC) program (activated 2006), which allows to spare more theeth than after conventional DC [11], and includes tight supervision and care by our dentists also in the periods during and post radiation

d) Finally, the fact of missing self-scoring xerostomia evaluation but from the very first IMRT patients [4] is a lack of information. Self-scoring has been recommended to be assess xerostomia [12]. Main reason not to focus any longer on this topic in the own patients were the detailed early investigations on xerostomia and salivary flow rates after IMRT by Eisbruchs’ group [13]. Their data fitted very well with the own clinical observations.

The presented evaluation revealed a low rate of 4% G3/4 persisting late term effects, reflecting the nowadays expectable level of treatment tolerance. The late effect rate as diagnosed 2 years post treatment completion was found representative and was unchanged at 5 years (Figure 3). Findings (Tables 2 and 3) compare well with published data from other centers on treatment tolerance in the IMRT/VMAT era. The comparability of IMRT late effects with that in published IMRT series is however somewhat limited due to different tumor cohorts assessed, different approaches to analyze and/or grade tolerance parameters (e.g. PEG dependence and/or pharyngeal dryness sometimes included in dysphagia rates, RON events not always graded, side effects related or not to subgroups at risk).

Treatment tolerance is clearly better nowadays compared with conventional pre-IMRT era prospective randomized trials. Reasons for this improvement are multifactorial; the main reason is the use of IMRT techniques allowing better sparing of organs at risk while delivering highly conformal tumor dose. The RTOG 9003 prospective randomized landmark trial compared normo-fractionation versus different other fractionation schedules in >1000 patients after conventional radiation techniques, and found grade >/=3 effects in 26.8-37.2% after a median FU of 23 months [14]. Similarly, the prospective randomized SAKK 10/94 trial investigated the effect of conventional technique hyperfractionated radiotherapy +/- concomitant cisplatin; the corresponding late effect rate was 111 events in 224 treated patients [15].

One of the important advantages of IMRT in oral cavity and oropharyngeal cancer is the reduced RON risk (1% G3/4 RON in our 'patients at risk’ cohort with oral cavity, lateral or central oropharynx cancer (oral cavity patients at highest risk (3%)), Table 2). The low RON rate in HNC patients is characteristic for the IMRT era and comparable with other published series [1,5,6]. None of our NPC patients treated with IMRT (n = 67 since 2002) was RON affected. RON rates reported in the SAKK 10/94 trial were 6% [15], in the RTOG 9003 trial 2.3% after a FU of median 23 months [14]. Comparison of the RON incidence after conventional radiation techniques during the period between 1980–1990 with 1990–1998 showed a decrease in the risk to a value of ~5% using three-dimensional (non-IMRT) techniques and hyper- or moderately accelerated fractionation [16].

PEG dependence and/or relevant dysphagia are often multifactorial (mucosal dryness, muscular dysfunction, tumor related anatomical defects) and were observed in hypopharynx, oral cavity or central oropharynx tumor patients; central oropharynx tumor patients were most often affected (3%). PEG dependence in oropharynx cancer patients was also described in a series from Memorial Sloan Kettering Cancer Centre (6/50) [17]. In the pre-MRT era, this rate was substantially higher (>20% permanent xerostomia and dysphagia in both study arms in the SAKK 10/94 trial [15], 10-15% dysphagia and 6-10% xerostomia in the RTOG 9003 [14], respectively). The low rate of persisting xerostomia grade 3 (3/51 NPC patients (6%), oropharyngeal and oral cavity cancer (<1%)) is in concordance with meanwhile numerous reports on parotid sparing in the IMRT literature [3,7,13]. The probability of severe xerostomia following radiation remains dependent on the dose and the anatomic distance of GTV to parotid glands and/or submandibular glands and the mucosal area included in PTVs. Considering the missing self-scoring of our patients, the assessed rate of G3/4 xerostomia may be somewhat higher.

Mucosal ulceration occurred in 28/593 (5%) patients with pharyngeal or oral cavity cancer (i.e. in patients with substantial areas of pharyngeal and/or oral mucosa included in the high dose planning target volumes, but in NPC patients), its location was always at the site of the former primary where also the boost dose was delivered. Most mucosal ulcers were painful, and clinically and radiologically hardly to differ from tumor persistence; its treatment was usually analgetic and/or antibiotic therapy. Early exclusion of local recurrence by biopsy is recommended. The onset of mucosal ulcer was diagnosed 0–18 months post IMRT completion (mean/median 5/3 months), comparable to published results [18]; complete healing took 1–12 months (mean 3.5).

In order to quantify the subgroup of risk for BPN, dose distribution plans of the first 100 consecutive patients out of 226 definitively irradiated patients with nodal disease have been reviewed: in 19/100 (~20%), the plexus was in part exposed to >65 Gy. No G3/4 brachial arm plexopathy was diagnosed in extrapolated ~40 patients at risk - in concordance with limited retrospective data from small HNC cohorts published on this topic [19].

Brain necrosis was not diagnosed in our NPC/sinonasal cohort as based on clinical and radiological FU assessments (22 cT4 NPC, 8 cT4 sinonasal cancer patients at risk). In all cases the dose was kept below 70 Gy delivered in 2.0 Gy/session to <1 cc brain.

Lhermittes’ sign in HNC is rarely reported, and was diagnosed in 4 patients. There was no obvious correlation with doses delivered (spinal cord <2.0 Gy/session in all patients, with a maximum dose of 46 Gy). Pak et al. prospectively reported on 73 affected patients and found an incidence rate of 21% which was related to higher spinal doses than in asymptomatic patients [20].

With regard to the question of cervico-vascular changes, no robust dose-volume related data are available so far but descriptions of vascular changes following conventional radiation from several small series [21].

## Conclusion

IMRT led to a high late term tolerance in the subgroup of loco-regionally disease free HNC patients, with only 4% persisting G3/4 effects. The onset of any G3/4 effects showed a plateau at 2 years.

The question of the cervical vessel tolerance in disease free long time survivors is still open and currently under evaluation at our institution.

## Competing interests

The authors declare that they have no competing interests.

## Authors’ contributions

GS drafted the article and created the underlying data base; GS, Cl, OR, YN, MB and CG performed the contouring and were in charge for the isodose plan reviewing, and treated the assessed patient cohort; CG and GS double-checked all contouring and isodose plans and are responsible for the IMRT-SIB radiation treatment schedules as defined/applied; BY assessed the dose distribution to the arm plexus region of the cohort; TR was involved in systemic therapy decisions for at risk patients and performed the chemotherapy in NPC patients; GH, MB, SS(4), RZ operated on these patients and performed the regular FU; SS(2) was in charge with dental long term rehabilitation of our patients. All authors read and approved the final manuscript.

## Supplementary Material

Additional file 1: Table S1 Late term effects related to diagnosis; red: persistent effects. Click here for file
