# Supplementary material for: Late term tolerance in head neck cancer patients irradiated in the IMRT era
Source: Radiat Oncol. 2013 Nov 5;8:259. doi: 10.1186/1748-717X-8-259 (PMC4229314; doi:10.1186/1748-717X-8-259)
Supplement: Additional file 1: Table S1 — Late term effects related to diagnosis; red: persistent effects. [file 1748-717X-8-259-S1.docx]

Additional file 1: Table S1: Late term effects related to diagnosis; red: persistent effects

* tumors of the ear, skull base, orbita

** persisting ulcer after postIMRT biopsy (no malignancy underlying)

*** tracheotomy due to postIMRT edema; persisting productive fistula after removal of tracheostoma

$ hypersensitivity to radiation (60Gy/2Gy, no systemic therapy) - delayed healing, persistent mucositis after >2y

° massive hypopharyngeal bleeding 4 months post IMRT, successful salvage surgery (ligation of external carotic artery)

°° 4x surgical nasal synechiolysis, plus 1x lacrimal duct stenosis
